# The making of urban ‘healtheries’: the transformation of cemeteries and burial grounds in late-Victorian East London^☆^

**DOI:** 10.1016/j.jhg.2013.05.001

**Published:** 2013-10

**Authors:** Tim Brown

**Affiliations:** School of Geography, Queen Mary University of London, London E1 4NS, United Kingdom

**Keywords:** Urban reform, Public health, Open space, Sanitary science, Amelioration

## Abstract

This paper focuses on the conversion of disused burial grounds and cemeteries into gardens and playgrounds in East London from around the 1880s through to the end of the century. In addition to providing further empirical depth, especially relating to the work of philanthropic organisations such as the Metropolitan Public Gardens Association, the article brings into the foreground debates regarding the importance of such spaces to the promotion of the physical and moral health of the urban poor. Of particular note here is the recognition that ideas about the virtuous properties of open, green space were central to the success of attempts at social amelioration. In addition to identifying the importance of such ideas to the discourse of urban sanitary reformers, the paper considers the significance of less virtuous spaces to it; notably here, the street. Building on Driver's work on ‘moral environmentalism’ and Osborne and Rose's on ‘ethicohygienic space,’ this paper goes on to explore the significance of habit to the establishing of what Brabazon called ‘healtheries’ in late-Victorian East London.

## Highlights

• The article explores the making of urban ‘healtheries’ in late-Victorian East London.
• ‘Healtheries’ here refers to burial grounds and cemeteries converted into parks and gardens.
• ‘Healtheries’ were important to attempts at improving moral and physical health.
• Emphasis is placed on the importance of habit and accessibility to contemporary debates.

‘An’ now, said the sweated one, the ‘earty man who worked so fast as to dazzle one's eyes, ‘I’ll show you one of London's lungs. This is Spitalfields Garden.’ And he mouthed the word ‘garden’ with scorn.^1^

Jack London took his description of this site, a garden that can still be found alongside Christ Church, Spitalfields in East London, further: ‘There are no flowers in this garden, which is smaller than my own rose garden at home. Grass only grows here, and it is surrounded by sharp-spiked iron fencing, as are all the parks of London town, so that homeless men and women may not come in at night and sleep upon it.’^2^ London's portrayal of this space was not limited to its size, aesthetic appeal, nor its defensive boundaries. He also caste a critical, though some would argue sympathetic, eye over the characters that he identified with it. These were not just homeless men and women locked out of this so-called green lung; they were the masses of ‘miserable and distorted humanity… [with] all manner of loathsome skin diseases, open sores, bruises, grossness, indecency, leering monstrosities, and bestial faces’ who had come to represent this part of the metropolis.^3^ This should come as little surprise; after all, such representations of East London's inhabitants as the literal embodiment of urban degradation and vice were reinvigorated in the 1880s and continued on and off well into the following century.^4^ Indeed, even more scientific social surveys such as Charles Booth's *Life and Labour* reflected social assumptions and prejudices of the day.^5^

**Fig. 1:**
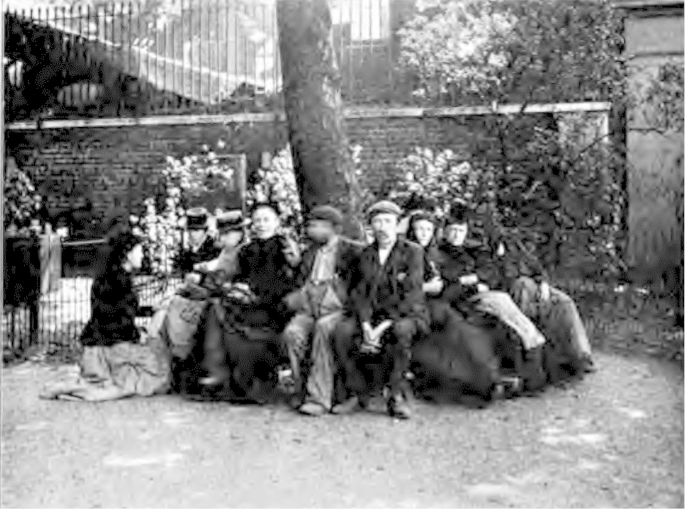
‘A Lung of London’. Source: J. London *People of the Abyss*, Norwood, 1903.

**Fig. 2:**
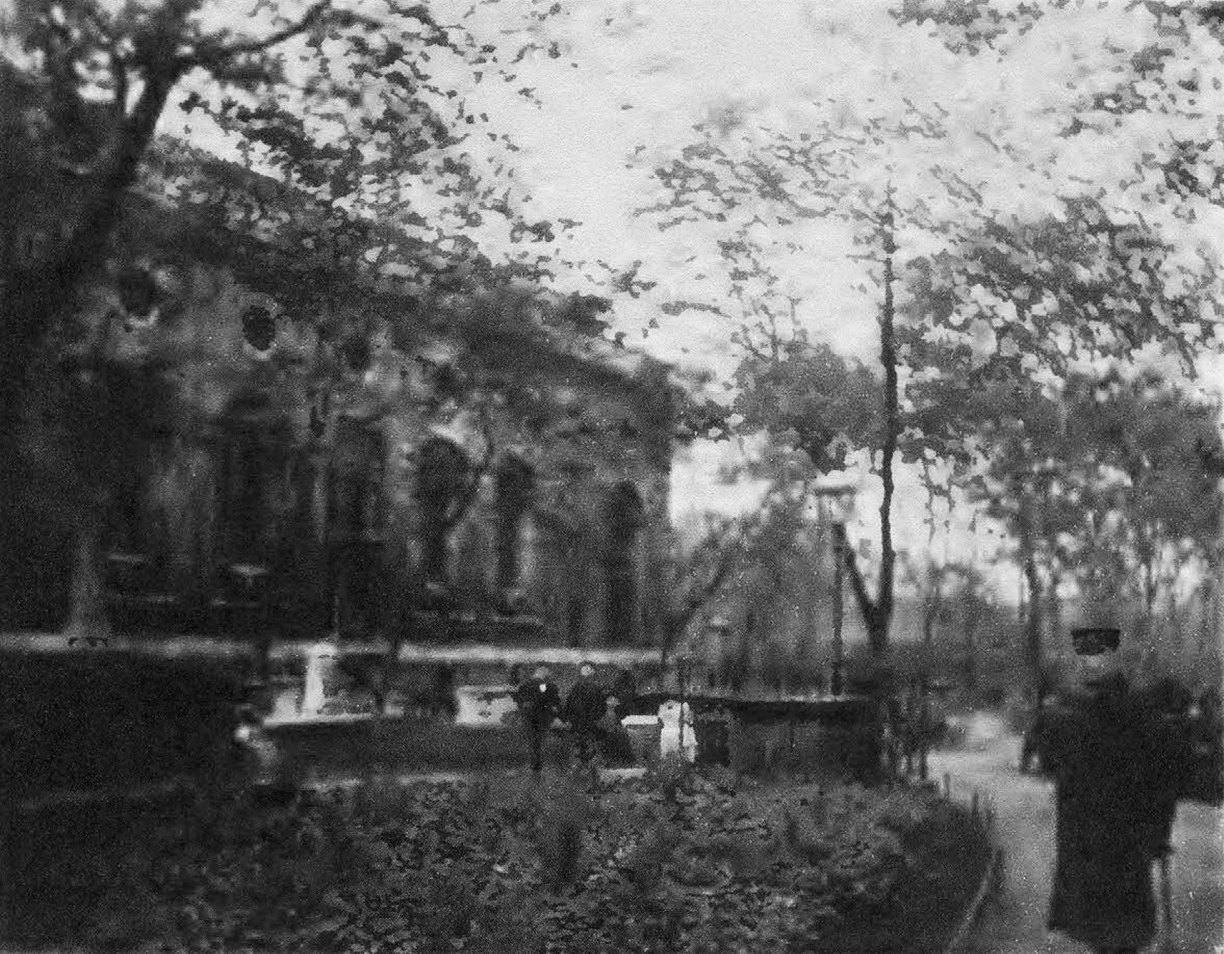
‘Christ Church Disused Churchyard, Spitalfields’.

Source: B. Holmes, Open spaces, gardens, and recreation grounds, in: Royal Institute of British Architects, *Transactions: Town Planning Conference*, *London 10–15 October 1910*, London, 1911, 491.

Jack London's account, and especially the images that accompanied it (see Fig. 1), is of interest here, though not primarily because of what it reveals about such representations of East London. It is rather because the garden that he was referring to was one of many disused burial grounds and cemeteries that had been converted into public gardens or playgrounds over the course of the previous 30–40 years. Moreover, if we look at sources other than London's *The People of the Abyss* quite contrasting images of the same space can be found. For example, a few years later Mr Basil Holmes, who was secretary of the Metropolitan Public Gardens Association (MPGA), offered a very different image of the garden at Christ Church to the participants of the Town Planning Conference, 1910.^6^ As Fig. 2 reveals, in this image there is evidence of the flowerbeds that London had suggested were absent. Further, the garden depicted is one that is neat and ordered, and whose occupants were kept under the watchful eye of a park keeper. The point here is not to dispute or challenge London's image, which depicts a different part of the same garden. Instead, it is to suggest that we can see two narratives at play here: one that, in Osborne and Rose's terms, sought to shine a light on the ‘dark continent’ of poverty and illuminate the degenerating effects of urban living especially on the abject poor, and another concerned with the remaking of urban space and on the possibilities for social amelioration.^7^

While London's account and others like it act as an important context which helps to explain the emergence of social ameliorism as a form of liberal governance, this paper focuses on projects that aimed to promote the moral and social improvement of the urban population through the production of ‘healtheries’. While the precise etymology of the term ‘healtheries’ is a little uncertain, it was applied to the International Health Exhibition held in South Kensington, London in 1884.^8^ This exhibition was dedicated to showcasing advances in the scientific study of health and education and in many ways reflected the progress that the sanitary science movement had made over the course of the century. Around the same time, Lord Brabazon, who, amongst other things, was founder and Chairman of the MPGA of which Basil Holmes was Secretary, utilised the term in one of his many publications on the subject of children's playgrounds. In a plea to the readership of *The Quiver* (an evangelical magazine directed at a largely middle-class audience), Brabazon highlighted their benefits in places such as Manchester and Salford: ‘If these towns have found advantage from the establishment of such “healtheries” in their midst, why should not all our large cities, and especially London, follow their example?’^9^

In making this plea for London to follow in Manchester and Salford's footsteps, Brabazon also drew readers' attention to work that his own organisation had begun. As he recorded, ‘[t]he 5th of May last was a red-letter day in the life of many a poor child living in the neighbourhood of the crowded district which surrounds the Borough Road, in the south of London. On that occasion a large playground, about one and a half acres in extent, provided with every equipment for healthy enjoyment, was thrown open to the children…’^10^ This ‘healthery,’ which resounded to the ‘joyful cries and laughter of thousands of merry boys and girls,’ was for Brabazon a far cry from the kind of ‘vice and misery, wretchedness and despair’ that he suggested was previously associated with the space. The motivation for this work, which in London also included the Commons Preservation Society (CPS), the Kyrle Society and the National Health Society amongst others, lay not only in the anti-urbanism of the period.^11^ As has been widely acknowledged, the provision of urban green, open space was a key concern of sanitary science from early on in its history.^12^ The term ‘healthery’ referred to technologies for promoting health and well-being in a variety of settings, including but not confined to playgrounds.

Lord Brabazon, latterly the Earl of Meath, is known to geographers as an arch-imperialist, a relatively minor actor in the work of social imperialism, and for his contribution to the re-imagining of London after the First World War.^13^ Although this paper picks up on the work of Brabazon where other geographical scholarship has left off, particularly with regards to his contribution to the reordering of already existing urban space, it does not focus on Brabazon alone.^14^ Rather, the paper positions Brabazon, the MPGA, and others concerned with the conversion of small urban spaces into parks and gardens within the wider context of Victorian sanitary science.^15^ Here, the paper engages with Foucault's consideration of space and the rationalities of liberal governance. As Foucault observed at various stages in his writing, the health and physical well-being of the population emerged as an essential objective of political power.^16^ Importantly, here, Foucault also pointed to the birth of a new set of technologies, from around the eighteenth century onwards, that sought to address the question of the population's health, and how to improve it, by addressing the problem of urban space.^17^

This concern has been picked up by scholars such as the British historian, Patrick Joyce, who, responding to Foucault's work on governmentality,^18^ suggests that the city emerged as a self-governing entity of liberal government during the period under investigation here and of central importance to this was the discourse of sanitary science, which was ‘characterised by a dynamic equilibrium between living organisms and their physical environment.’^19^ This notion of dynamic equilibrium, of a deterministic relationship between bodies and their environments, is of particular significance because the problem of population health, especially as it related to the poor health found in Britain's major cities, was articulated as being the problem of specific sites within the city and many of the solutions that emerged were spatial ones.^20^ Further, as Osborne and Rose note in reference to their notion of ‘ethicohygienic space,’ ‘[t]he spatial relation of citizen to habitat,’ Joyce's dynamic equilibrium, ‘was turned into one that can and should be governed.’^21^

While the spaces upon which this paper focuses on are relatively small in size, and the ambitions of the main protagonists are modest in comparison to the kinds of utopian vision that had come earlier in the century with, for example, Benjamin Richardson's *Hygiea: A City of Health* or later on with the Garden Cities movement,^22^ they do form an important element of this form of liberal governance.^23^ As the paper demonstrates, a central feature of this discourse is the lack of provision for accessible open, green space in the ‘rookeries’ and ‘swamps’ that Driver describes in his discussion of nineteenth-century moral environmentalism. A particular concern here was that these inhabitants, especially the elderly and the young, could not benefit from the health enhancing and socially improving qualities of such spaces, nor, importantly, could they develop the kinds of healthful habits that ameliorationists sought to promote.^24^ In the light of this, the conversion of former burial grounds and cemeteries into playgrounds and/or publically accessible green space, the kind of work the Gilbert refers to as ‘piecemeal developments’ of the existing urban fabric were regarded by many as essential to improving the physical and moral health of the population.^25^ While this paper does not focus on the grand strategies associated with the remaking of urban space during this period it does focus on sites that might be regarded, in Foucault's terms, as the ‘little tactics of the habitat’ associated with urban sanitary reform.^26^ Put differently, the paper contributes further understanding of the ‘motley of inventive projects’ that were implemented to induce the ‘rights kinds of habits’ in the darkest spaces within the city.^27^

This paper addresses these stated ambitions through reference to extensive archival research conducted primarily into the work of the Metropolitan Public Gardens Association.^28^ Additional material relating to the wider context within which this ameliorating work was conducted is drawn from contemporary newspaper accounts, popular and scientific journals, and pamphlets. The paper proceeds in three further substantive sections that address: firstly, the rhetorical links between the MPGA and other key organisations, notably the Kyrle society, and wider debates relating to sanitary science; secondly, the work of the MPGA in securing open, green spaces in East London; thirdly, the importance of location and habit to this discourse of social amelioration.

## Places to sit, spaces to play

Social science is described by Eileen Yeo as starting its life as a ‘science of the poor.’^29^ From the 1830s it heralded wide-ranging social enquiries into the condition of the working classes whose lives had been so transformed by the processes of industrialisation and urbanisation. For Hamlin, this is a crucial period that helped to define how social science would, in the form of an emergent ‘sanitary science’ or public health, respond to the particular problem of the health of the poor.^30^ Referring especially, though certainly not only, to the influence of Edwin Chadwick, both prior to and after the publication of his *Report on the Sanitary Condition of the Labouring Population of Great Britain*, 1842, Hamlin suggests that in following arguments for the environmental causes of disease sanitary science retreated ‘from the problem of the diseased body’ to the increasingly more abstract levels of the ‘house, street, town, region.’^31^ The significance of this emphasis on space rather than on bodies was picked up by Driver who noted in 1988 that underpinning social science was a particular form of medico-moral environmentalism, one that mapped behaviours on to particular kinds of (usually urban) space.^32^ Under this schema, or what Osborne and Rose refer to as a diagram,^33^ of the city, the distribution of health and virtue or disease and vice was to a large extent argued to be closely associated with, even dependent upon, the influences of the physical environment.

Where this led sanitary science, and where this leads this paper, is to forms of intervention, the motley of inventive projects mentioned above, that sought to counter the corrupting and degenerating effects of the city on the bodies of the poor in particular by improving the environments in which they lived out their lives. Of course, the passage from Chadwick's Report to the emergence of a wide-ranging and influential sanitary reform movement in the latter quarter of the nineteenth century was not a straightforward one. The years that followed the publication of the Report were packed with official and unofficial responses to the problem of urban health, with Sir Robert Peel establishing the Royal Commission on the Health of Towns and Populous Districts in 1843.^34^ The reports produced by this Commission, which were heavily influenced by Chadwick and presented to parliament in 1844 and 1845, firmly located the causes of ill-health and high mortality in the most densely packed and unsanitary parts of Britain's major cities.^35^ With the help of organisations like the Health of Towns Association, formed in 1844, the Commission resulted in the passing of the Public Health Act, 1848. As Hamlin notes, this was crucial to the establishing of space rather than bodies as the focus of sanitary science.^36^ However, Britain was not, at this time, ready for the kind of interventionist strategies that were being sought by sanitary science and proposed reforms, especially those in the Public Health Act, 1848, were subject to considerable delay and prevarication.^37^

While this may be so, the significance of this early period in the history of sanitary science, to this paper at least, is that it united middle-class concerns for local amenities with ‘evangelical visions of a coming world of morality, decency, and cleanliness.’^38^ As Driver notes, the watchword for this kind of work was ‘improvement’ or social ameliorism and, although reforms to the urban fabric of cities came up against resistance, organisations such as the National Association for the Promotion of Social Science, which included a health department from its establishment in 1857, helped to awaken the population more generally to the sanitary gospel.^39^ As Hamlin observes, ‘sanitation was to be synonymous with doing good.’^40^ The influence of this heady mix of sanitary science and Christian evangelism was, perhaps, most keenly felt in Britain's municipal cities in the mid-century.^41^ However, as Wohl suggests, the ‘great agitation of the 1880s,’ prompted as it was by Andrew Mearns' *The Bitter Cry of Outcast London*, saw advocates of sanitary reform operating in a much more favourable atmosphere in London. It is from within this atmosphere that work on transforming disused cemeteries and playgrounds into parks and gardens emerged.

The promotion of urban spaces for health and recreation mirrored these general shifts in the sanitary reform movement,^42^ although the recognition of the need for spaces in towns and cities in which the middle and working classes could pursue ‘rational recreation’ came much earlier with the Report of the Select Committee on Public Walks in 1833.^43^ That a connection was being made between health and access to open, green space during this period can be seen from the following paper read by its author, a Miss M.J. Vernon (an ordinary member of the MPGA) at the Social Science Congress at Cheltenham, in 1878 and published in *The Sanitary Record*. In the paper, Vernon spoke about the value of such spaces for the promotion of health: ‘The question… of providing in town public parks is one of urgent importance, not only as a method of controlling in some measure the density of population… but also as providing places for healthy exercise and health-giving recreation.’ Moreover, she set out her vision of what an ‘ideal public park’ would look like: ‘there should be some meadows trim, with daises pied,’ and shady walks and rockeries; borders filled with the flowers poets sing of, shrubs and flowering trees.’^44^

To this admittedly rather idyllic image, Vernon added the need for bathing pools, cricket grounds and ‘space for active games of all sorts,’ cafés to ‘combat the inevitable alehouse outside’ and music. In this way, she was clearly talking to a much broader sanitary science discourse which recognised that parks and gardens were spaces for social and moral improvement as well as sites for the promotion of physical health. In other aspects of Vernon's paper it is apparent that her engagement with these ideas went much deeper than mere rhetoric; for example, her reference to density of population echoes Benjamin Richardson's *Hygeia* and later in the paper she refers to her use of statistics provided by William Farr. Importantly, here, there is also an acknowledgement that much smaller spaces than those mentioned above were needed for the urban poor: ‘Parks are not all we want. They must of necessity be far apart, and we should have open spaces near together. Parish gardens they might be called, where the invalid and aged would creep out of the close alley and dreary court to bask in the sunshine; where the child-nursemaid could safely be left to mind the little ones, and the older children would enjoy happy, hearty play. How our decent poor would hail such gardens.’

As Driver highlights, the work of social amelioration relied upon the construction of a dialogue between the immanent virtues of open, green spaces such as parks and gardens and the immanent dangers of the overcrowded courts and alleys, streets and gutters of the city. That the former promote health was not linked to particularly clear understanding of causality, rather it was based upon statistical associations produced by the likes of William Farr and replicated in accounts such as Vernon's.^45^ Further illustrations of this dialogue can be found elsewhere. For example, Octavia Hill, influenced by the ideas of John Ruskin, emphasised the importance of colour and light as well as of space and fresh air.^46^ Commenting on the need for ‘[p]laces to sit in, places to play in, places to stroll in, and places to spend a day in,’ Hill referred to the need to ensure that the former amongst these places were ‘very near the homes of the poor’ and that they should be ‘pretty and bright.’ Moreover, Hill draws the kind of contrast that Driver suggests is so central to this discourse:

I go sometimes on a hot summer evening into a narrow paved court, with houses on each side. The sun has heated them all day, till it has driven nearly every inmate out of doors. Those who are not in the public house are standing or sitting on their doorsteps, quarrelsome, hot, dirty; the children are crawling or sitting on the hard hot stones till every corner of the place looks alive, and it seems as if I must step on them, do what I would, if I am to walk up the court at all… Sometimes on such a hot summer evening in such a court when I am trying to calm excited women shouting their execrable language at one another, I have looked up suddenly and seen one of those bright gleams of light the summer sends out just before he sets, catching the top of a red chimney-pot, and beautiful there, though too directly above their heads for the crowd below to notice it much. But to me it brings sad thought of the fair and quiet places far away, where it is falling softly on tree, and hill, and cloud, and I feel as if that quiet, that beauty, that space, would be more powerful to calm the wild excess about me than all my frantic striving with it.^47^

Here, we also see Hill make the connection to the value of disused burial grounds and cemeteries: ‘The most easily available places would be our disused churchyards. I have myself no fear that the holy dead, or those who love them, would mind the living sharing in some small degree their quiet.’^48^ Pointing to one such place, ‘a small, square, green churchyard in Drury Lane,’ Hill suggested that they should be made ‘bright, pretty and neat’ and that, if they were so, they would help overcome the oppressive character of the court: ‘a tiny cloistered court of the same size will give a sense of repose; and colour introduced into such enclosed spaces will give them such beauty as shall prevent one from fretting against such boundaries.’ Hill's focus on disused churchyards was taken up by others. For example, in a story published in *The Graphic*, one of the many journals and newspapers that began carrying significant numbers of stories on issues relating to sanitary reform,^49^ it was noted that it was ‘almost impossible to imagine any more desolate and depressing a spectacle than an unused and uncared-for metropolitan burial ground’ and that all the ‘neglected, and as it would seem forgotten, resting-places of the dead might, as we have said, with but little trouble, be made conducive to the comfort of the living.’^50^

As these various accounts suggest, there is often little explicit reference to the ways in which access to green, open spaces actually promotes health. There was not the same demand for an evidence-based approach that is so important today.^51^ Rather, what we find is the identification of particular kinds of habit, good and bad, with the immanent properties of urban space. Where habits needed to be improved, at least in the minds of social improvers, the solution was not to explicitly act upon the bodies of the urban poor but rather on the spaces they inhabited: ‘The planting of a few trees, the making of a few flowerbeds, and the construction of a few gravel walks, with seats long them, would convert each one of these dreary solitudes into a grateful oasis amid the almost endless wilderness of metropolitan bricks and mortar.’^52^ As Osborne and Rose suggest, what we see at play here is not the elimination of moral danger through ‘grids of domination’ but attempts to produce a kind of ‘regulated, civilised subjectivity’^53^; here, through the securing of urban healtheries that were within easy access of those most in need. It is upon the process of securing these spaces that the paper now focuses.

## Securing urban healtheries

**Fig. 3:**
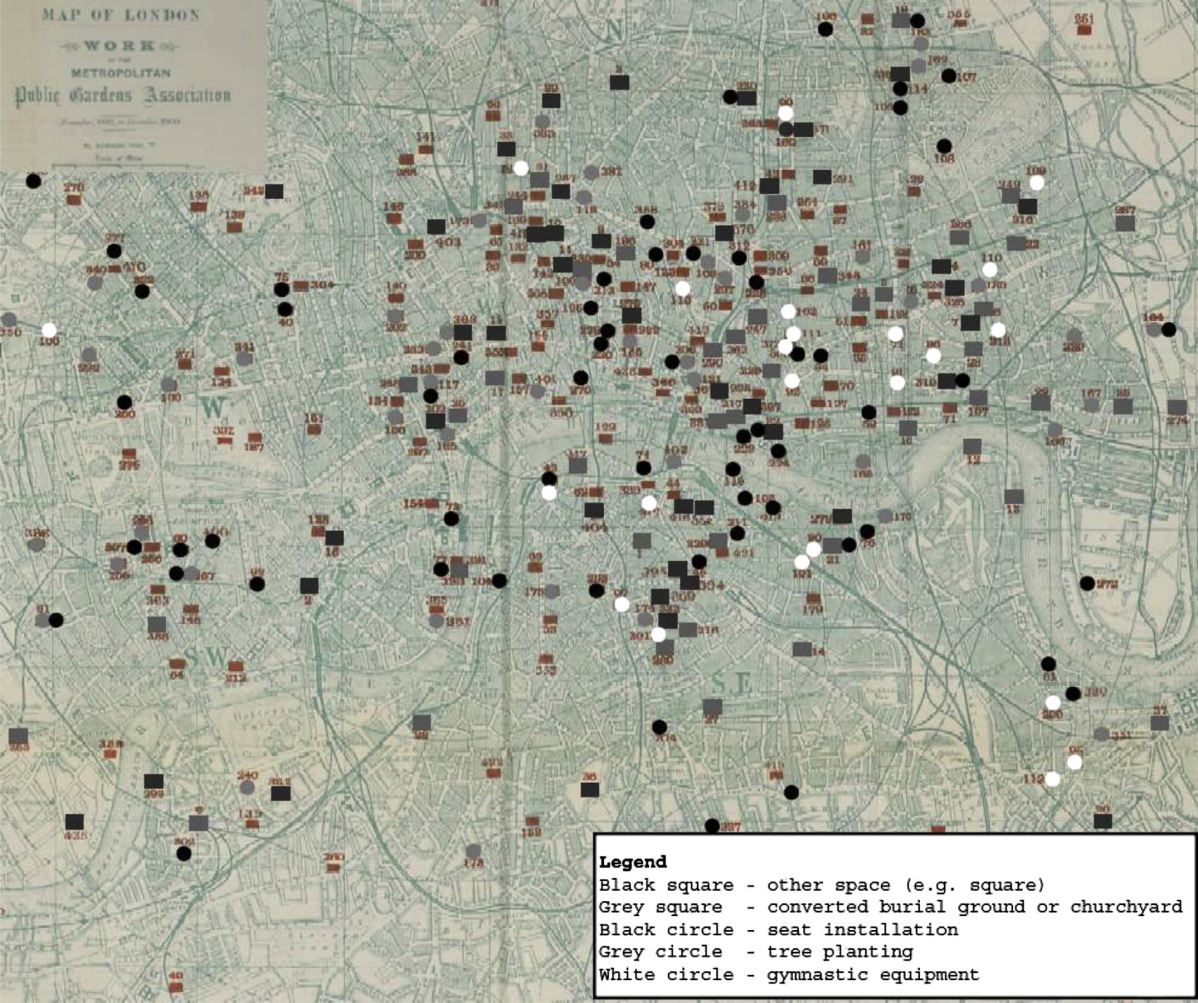
Work of the Metropolitan Public Gardens Association, 1882–1900.

Source: MPGA, 18th Annual Report.

While Octavia Hill continued as a key advocate of open spaces throughout the period,^54^ Malchow suggests that ‘[m]ore serious ameliorationists turned elsewhere.’^55^ The MPGA, or the Metropolitan Public Garden, Boulevard and Playground Association as it was originally named,^56^ was where many turned. Established in 1881 according to Brabazon, the inaugural meeting took place in November of the following year at Lancaster Gate, London and while it shared many of Hill's ideas the organisation was far more effective in terms of bringing them into being (see Fig. 3).^57^ There were at this first meeting of the MPGA a mixture of aristocrats, barristers and solicitors, East London clergy and middle-class women, the latter of whom were centrally involved in the sanitary reform movement from the mid-century onwards.^58^ Brabazon, in his opening address to them, remarked that its formation was prompted by a number of concerns: the lack of ‘recreation and breathing room’ in the city's most densely populated areas, the inaccessibility of London's already existing parks to the urban poor, and by the ‘need of playgrounds for the young.’^59^

Before looking in more detail at the ameliorating work of the MPGA, it is worth exploring the contribution that Brabazon made to contemporary debates on the subject. Like Hill, Brabazon was a prodigious author: he published widely in journals such as *The Nineteenth Century* and the *National Review*, contributed a considerable number of letters to newspapers such as *The Times*, and also published many books including *Social Arrows* (1886) and *Prosperity or Pauperism* (1888). In one of the earliest of his papers, which appeared in *The Nineteenth Century* under the title ‘Health and Physique of our City Populations,’ Brabazon established his credentials as an imperialist, advocate of physical culture, and as a social reformer.^60^ Hailing the ‘strength of body’ and ‘undaunted courage’ demonstrated by Britain's imperial explorers, Livingstone, Burton and Speke, he remarked that this physicality, if not the endurance, was shared by ‘almost all Englishmen’: ‘It is hardly possible,’ he noted ‘to take a stroll of a Saturday afternoon in the well-to-do outskirts of a populous town without seeing a game, and it may be several games, of football in winter, and of cricket in summer, being engaged in by a large number of young men who, during the rest of the week, have been occupied in business pursuits.’^61^

Here, Brabazon pointed to what he regarded as a new English muscularity: ‘[t]he effeminate shop-clerk… has developed into the stalwart volunteer, the oarsman, the bicyclist.’ What connected all of these examples of health and vigour was their greater ability for ‘getting into the country.’ However, while he acknowledged that aristocratic and middle-class men were able to resist the ‘deleterious influences of civilisation and of city life,’ in contemporary terms they were, perhaps, resilient to it, the same was not true of the urban poor. This latter point is a feature that regularly emerged in Brabazon's writing, especially as it related to ideas of national vitality: ‘though it may be difficult to prove by statistics… it is only necessary for an intelligent man or woman to walk through the slums of our great towns in order to assure himself or herself, beyond all question or doubt, that the physical condition of the people in these crowded districts is, to say the least, unsatisfactory.’^62^ As with the discussion of other examples of this discourse, the immanent, and here harmful, qualities of city life were argued to have acted upon the bodies of the poor, leading Brabazon to question, ‘[d]o our athletes, our sportsmen, our travellers, our mountaineers, issue from the crowded lanes of overgrown cities?’^63^

Brabazon's ideas regarding the health promoting properties of green space were unsurprisingly deeply engrained in the work of the MPGA; here reflected in their statement of objectives, which stated that the city, at least those parts of it that were devoid of parks, gardens, and playgrounds, was not a place where ‘bodily functions can have full and natural play, where bone and muscle in the young man may be developed, and where constitutions are able to ward off disease and decay.’^64^ However, this should not be taken to mean that either Brabazon or the MPGA were primarily concerned with attending to issues of physical degeneration. As the statement on objectives went on to clarify: ‘though… no panacea for human suffering and misery’ access to open spaces provided ‘a retreat from the suffocating, sickly smell of the dwelling, where the eye may be refreshed with a glimpse of nature, and where the ear may rest from the jangle of voices and the rush of traffic.’ So, as with Vernon, Hill, and others writing on this aspect of sanitary science green, open spaces were argued to revitalise the human spirit as well as to improve the vitality of those living in the city's overcrowded and densely populated spaces.

While we might regard this language as simply rhetorical, the MPGA contributed much more to the question of social amelioration than words alone. Between 1882 and 1900 the Association was involved in over 400 different projects: including, lobbying for changes to existing legislature, planting trees and providing park benches (by 1893 it was estimated that some 2500 trees and 1200 seats had been either planted or placed across the city),^65^ and converting over 100 disused burial grounds and cemeteries and other spaces (notably squares) into publically accessible gardens and playgrounds (see Fig. 3). Additionally, they encouraged schools to open their playgrounds to local children on Saturdays, made donations to support the equipping of gymnasia (over 30 gyms, mostly located in parish churches, received donations amounting to nearly £850), and supported other associations or organisations in their work in laying out gardens, building gymnasia, playgrounds, and swimming pools (over £1600 was donated for these purposes).^66^ Amidst this wide-range of work, all of which was dedicated in some way or other to social amelioration, the provision of green, open spaces was a priority for the MPGA from the outset:

His Lordship in opening the proceedings stated that he had been actively concerned in creating the meeting with a view to establishing an Association for promoting the laying out for the benefit of the people of the Metropolis every morsel of land available for the purpose, because he was persuaded of the chiefest need for the inhabitants of the poorest and most densely populated parts of the Metropolis in more recreation and breathing room: and that the only way in every wise to meet this pressing need is the formation of a Society which should have for its main object the giving to the people gardens and to the children playgrounds.^67^

As with Octavia Hill and her sister Miranda, who had read a paper to the National Health Society on the subject, Brabazon and the MPGA recognised the potential value of the city's disused burial grounds and cemeteries in this regard.^68^ As was noted at their inaugural meeting, such spaces were amongst those that ‘might be gratuitously acquired and utilised as gardens or playgrounds.’^69^

At the MPGA's second meeting, held in December 1882, members of the Association were appointed to watch over different districts within the city and to correspond with the Executive Committee on sites that should be targeted for conversion to gardens or playgrounds. Furthermore, they were encouraged to watch for signs of development upon them.^70^ The Association's focus on burial grounds and cemeteries was enhanced considerably by the passing of The Disused Burial Grounds Act of 1884, which forbade building on former burial grounds except for extending a church, chapel, meeting house, or other place of worship. As Malchow notes, this ‘at a stroke opened the way to what became the most vigorous and successful’ of their activities because it destroyed the commercial value of the land.^71^ Within East London, defined here quite broadly to include the contemporary boroughs of Hackney and Tower Hamlets to the west and Barking to the east, the first former burial ground or cemetery to be converted to a publically accessible garden by the MPGA was St. Bartholomew's churchyard in Bethnal Green. The churchyard, a half-acre site situated at the back of the church, was laid out and opened to the public on May 4th 1885 after nearly 2 years of negotiation between the MPGA and the Vestry of St Matthew, Bethnal Green, which was the body responsible for parish affairs.

After St. Bartholmew's came a succession of other similar gardens laid out by the MPGA: East London Cemetery, Mile End Old Town (July 1885), West Hackney churchyard (October 1885), St. Paul's churchyard, Shadwell (May 1886), St. John-at-Hackney churchyard (June 1886), Holy Trinity churchyard, Mile End (May 1887), St Anne's churchyard, Limehouse (October 1887), and Trinity Chapel disused burial ground, Poplar (June 1888). After this initial burst of activity, many more such spaces were opened to the public from the 1890s onwards. Perhaps, the most significant of these later gardens was Victoria Park Cemetery or Meath Gardens, Bethnal Green. The MPGA first enquired about the possibility of converting this disused cemetery in June, 1885. In a letter sent to the trustee of the site, the Reverend J.B.D. Butler, a case for conversion was set out; one that described the nature of the locality and emphasised the ‘immense boon’ to the health and enjoyment of the inhabitants that such a garden would bring.^72^ Like so many of the sites already discussed, there was considerable delay in acquiring and then laying out this particular space. The initial enquiry was clearly unsuccessful, as a report to the MPGA Committee in December 1890 noted that a delegation sent to look at the cemetery had found it ‘in a great state of neglect, many gravestones being broken and the pieces scattered about, several graves sunk in, and one actually open…’^73^ However, the land was eventually handed over to the Association for landscaping in 1893.

**Fig. 4:**
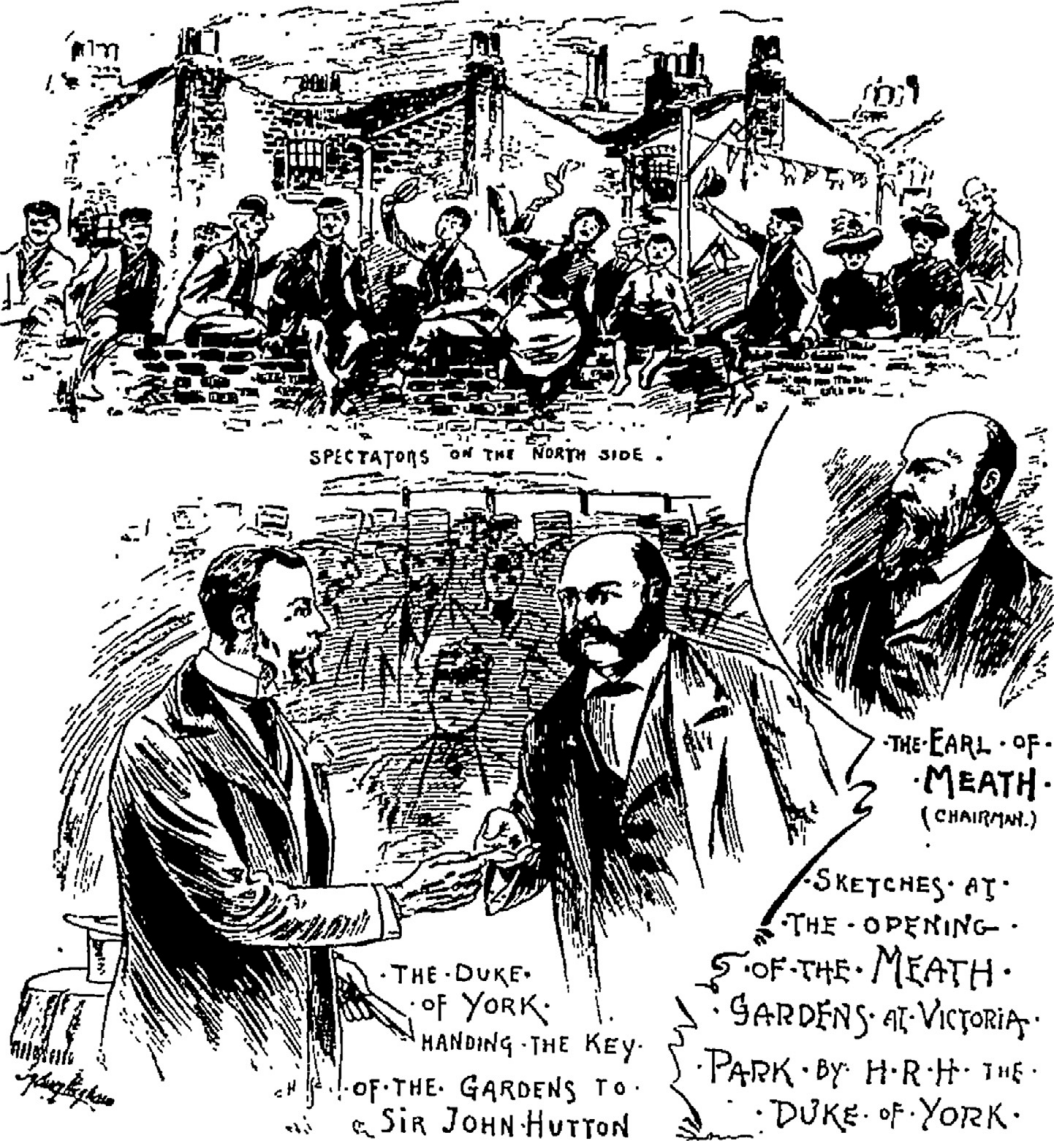
‘H.R.H. The Duke of York Opening Meath Gardens, Bethnal Green’.

Source: *Penny Illustrated* 1894.

As with the majority of sites converted by the MPGA, Fanny Wilkinson, the Association's honorary landscape gardener, was responsible for the entire process. As Wilkinson stated in a rare interview with *The Women's Penny Paper*, she had been trained at Swanley Horticultural College and her role was to ‘design, draw out plans, and see them executed.’^74^ Little of the actual detail of this conversion process remains, although the minutes of the MPGA reveal that some 30 men were employed to convert the 11-acre site into gardens and playgrounds under Fanny Wilkinson's charge. Some additional detail is provided by John Sexby in his contemporary account of the history of municipal parks and gardens in London.^75^ In this Sexby provides before and after photographs of the site, these were a common feature of the work of the MPGA but only a few images remain, and a short written record of its primary features: ‘The greater portion of the ground is laid out as a garden, and the remainder is devoted to two large playgrounds for boys and girls, fitted with swings, see-saws, and gymnastic apparatus.’^76^ The former cemetery was eventually opened as a public garden and playground in 1894 by the Duke of York; and to great fanfare (see Fig. 4).^77^ As a report on the opening ceremony, published in the *Penny Illustrated*, stated, the gardens, once regarded as ‘dismal and squalid’ and, according to Sexby, the ‘resort of the loafers and roughs of the East End,’ had been turned into a ‘fair and wholesome pleasure resort, and has become a cheerful and healthful ornament, instead of a shameful blot to the neighbourhood of Bethnal Green.’^78^

## Embodying good habit

The MPGA and organisations like it clearly played an important role in securing open, green spaces that were publically accessible to those living in London's most densely populated districts. Whether or not these small-scale interventions had the desired impacts on health and vitality is open to question as there were no systematic attempts to evaluate their effectiveness. Though for Brabazon, as for Octavia Hill and others concerned with social amelioration, there could be little doubt that they did: ‘Does the young man never long for some quiet corner amid trees, and surrounded by flowers and water and the beauties of nature… Does the thought never enter the working class matron's mind that she would be the happier and the better could she occasionally, on a fine summer's day, take her work or a book to a neighbouring garden… Would the working man not prefer to discuss his dinner, his pipe, and his newspaper seated on a comfortable bench away from the scene of his labours, its dirt, and its commonplace surroundings?’^79^ As this suggests, widely circulating ideas within the sanitary movement about the importance of sunlight, fresh air, and access to nature were central to arguments being made for the need to provide these spaces.

Yet, as discussed at the beginning of this paper, of crucial importance to the MPGA, as for others involved in providing open spaces for the poor, was the spatial relation between citizen and habitat. This was a question that vexed ameliorationists because they recognised the need for such spaces to be convenient to those residents whose physical and moral health they sought to promote. An article written by Walter Besant for the *Pall Mall Gazette* under the title ‘The social wants of London,’ highlights the importance of this point perfectly. In the article, Besant encouraged the readership to reflect critically on the city's provision of parks and gardens: ‘he who considers the map [of London's green spaces] becomes aware of one or two facts which disturb his complacency… most of [the parks] are practically inaccessible to the population.’^80^ Besant took his discussion of inaccessibility further, highlighting that there were in essence ‘no open spaces’ that were close enough to the homes of those who were most in need of them. Thus, despite an acknowledgement that London as a whole was a relatively green city, the main issue for Besant and by association for places like East London, was how to provide spaces that were accessible to those who according to the sanitarian logic were in most need of them.

In order to justify his emphasis on this question, Besant pointed to the important issue of habit and its relevance to the ambitions of the sanitary reform movement: ‘Consider: a park is really available for the purpose of habitual resort,’ as a ‘garden in which fresh air, flowers, and trees may be enjoyed, without fatigue and after the day's work,’ only when it is ‘within a mile or three-quarters of a mile.’^81^ Any distance greater than this, and even less for younger children and the elderly, was considered by Besant to be ‘too great to make its use habitual.’ Besant's concern with the location of these spaces was, as noted earlier in this paper, shared by others. Moreover, it was a point that Brabazon, then the Earl of Meath, returned to in a paper written a few years after Besant for *The Nineteenth Century* in 1893: ‘The ideal of the writer of this paper is that a small garden or a children's playground divided into two portions, one for boys and one for girls, both supplied with gymnastic apparatus and appliances for suitable games… under care and supervision of special attendants, should be opened and maintained by the municipal authority in every large city within a quarter mile's walk of each working or middle-class home.’^82^

The modification of disused burial grounds and churchyards that were close to the homes of the urban poor, though as Brabazon indicates not their homes alone, was clearly associated with the desire to make the use of such individually and socially beneficial spaces habitual.^83^ While limited, the evidence provided by caretakers suggests that this was achieved at least to an extent. For example, with regards the disused burial ground in St. Bartholomew's Church, Bethnal Green it was noted that ‘the garden is always frequented by adults and children’ and the East London Cemetery, Mile End, described as a ‘neglected wilderness of rank grass and broken tombstones,’ before being laid was reported as being ‘much frequented by the children of the crowded locality.’^84^ However, the question of habit arises not only in terms of habitual use but also in terms of the ways in which different spaces within the city were seen to promote different types of habit; bad and good. Indeed, Besant drew on middle-class anxieties associated with the habits promoted by the city's streets as a means to promote the kinds of work that the MPGA and others were doing:

There are, therefore, for all these people no gardens or pleasant walking places for them; there are no playgrounds; there are no open spaces where they can sit down and talk at their ease; there is nothing at all but streets. The children play in the streets; whenever it is not raining they live in the streets; the old people take their walks abroad in the streets; the working men in the evenings lounge and smoke their pipes in the streets. There is no quiet for anybody; no rest from the continual din, traffic, and bustle of the crowded streets; no escape from the suffocating air in summer; no place where the children can play without danger of being run over; none where they can go through the discipline and drill of organised play; there is no chance of observing the approach of spring and the glory of summer.^85^

Similar concerns about the street as a site of threat to physical and moral health were expressed elsewhere. For example, in the 1870s Octavia Hill noted that it was undesirable for children ‘to play in alleys and in the street, learning their lessons of evil, in great danger of accident, and without proper space or appliances for games.’ In a similar vein, an article published in *MacMillan's Magazine* stated that ‘[r]espectable parents, rightly and naturally, will not let their children run loose about the streets, where there are so many dangers, physical and moral.’ Where both of these stories emphasised the moral dangers of the street, Brabazon, in an essay published in *The Quiver* commented on other dangers: ‘the gutter, the close alley, or the dangerous crowded street are the only places where they can play and stretch their limbs,’ and if children ‘encroach on the pavement they become a nuisance to passengers, and are arrested by the police, whilst if they play in the road they are in constant danger of their lives.’

There is much more that could be said about such concerns over the disordered nature of the street and of the ways in which Victorians began reordering their material features and the behaviours of those who occupied them as they sought to bring order to the city.^86^ However, the key point here is that this discourse relating to the immanent dangers of city spaces such as the street was mobilised in part to promote the importance of gardens and playgrounds to the healthful development of children; that is, to the promotion of good habits. As the minutes of the MPGA reveal, there were considerable concerns over the nature of the children, and of course adults, that occupied the districts of East London. Many of the former burial grounds and churchyards that the Association converted had to be ‘defended,’ sometimes with barbed wire, from young people who were regarded as a threat either to their material features, there were many reports of flowerbeds being destroyed and flowers being stolen, or to their tranquillity.^87^ Indeed, in a letter sent to the Limehouse Board of Works in June 1884 the MPGA acknowledged that there were concerns that ‘the young people living in the neighbourhood would destroy these grounds if laid out as gardens, and by their general conduct render them totally unfit for respectable people to frequent, and a nuisance to the neighbourhood.’^88^

As these examples illustrate, there was an acknowledgement that the bad habits engendered by even a short amount of time living in these neighbourhoods required more to be done than mere modifications to the urban fabric of the city. Put differently, the MPGA, like other societies, recognised the need for constant surveillance if order was to be kept in the spaces that they provided and if their ameliorating effects were to be felt by young and old alike. It is for this reason that caretakers were provided by the MPGA; not only within the smaller gardens attached to churches but also in the much larger playgrounds. As Brabazon indicated in his 1893 essay in *The Nineteenth Century*, the importance of the caretaker, often as Fig. 2 illustrates in uniform, was a lesson that had been learnt by the MPGA: ‘Experience has shown that in Great Britain public playgrounds must never be left without a caretaker, and should be closed after dark; but if these precautions are taken, and if in rough districts special aid be given the caretaker for the first two or three weeks after their opening, no further difficulty need be anticipated.’^89^

While the caretaker was introduced to maintain order in gardens and playgrounds, the MPGA like other reform organisations did not rely solely on their influence to cultivate the kinds of habits that they sought to instil. For example, as others have noted of the playgrounds movement more generally, such spaces involved the introduction of technologies that were designed to promote normative ideas of physiological and psychological health.^90^ The simple positioning of park benches and trees in the gardens and playgrounds might be offered as examples of this; after all, these were introduced to slow people's passage through them, to provide shade in the summer and shelter in the rain, and to allow the calming and relaxing benefits of these spaces to be experienced. Then, as now, they were also introduced to encourage habitual use. However, perhaps more obvious examples can be found in the gymnastic equipment that was provided by the MPGA. As Brabazon indicated, he had been influenced by the Manchester and Salford Sanitary Association in 1884 and the equipment that was introduced – which in the larger sites such as Meath Gardens was split between boys and girls playgrounds and included giant strides, climbing masts, horizontal and parallel bars, swings, jumping-boards, swings, skipping-ropes, and horizontal ladders, swinging rings – had as much to do with disciplining the body and promoting good habits as it did with providing for the enjoyment and pleasure of the young.

Indeed, while extending a little beyond the main scope of this paper, it is apparent that Brabazon's concern for the promotion of physical training in the nation's youth had a broader influence on the work of the MPGA.^91^ Specifically, the Association established a sub-committee to focus on physical training in 1886 to work with the National Health Society (whose Chairman, Ernst Hart editor of the *BMJ*, was Vice-Chair of the MPGA), the Kyrle Society, and with two Manchester philanthropists, Mark Phillips and Thomas Horsfall, associated with the Manchester and Salford Sanitary Association to promote physical training.^92^ Moreover, a gymnastic instructor was employed at some of its larger playgrounds from November 1890 and by 1892 this included the grounds of the People's Palace, Mile End and the playgrounds in Victoria Park; the former of which had received funding from the MPGA for the provision of gymnastic equipment.^93^ As a report from the gymnastic instructor suggests the classes, which ran on Saturdays, appear to be well attended, with 381 girls and 316 boys attending in February 1892. As in Gagen's discussion of the American physical education movement that emerged at the same time, the aim here was not only to train the bodies of these young people but also to secure in them the kinds of moral behaviour that such exercise was believed to promote.^94^

Such instruction did not appear to take place in the smaller playgrounds or gardens discussed above; here, however, it is likely that other forms of instruction were in place. As records from contemporary magazines suggest, and as others have noted, the promotion of gender-appropriate play was fostered in many such playgrounds.^95^ As the author of an essay in *Macmillan's Magazine* suggests, ‘[p]lay is known to be indispensable for the children of well-to-do parents, and arrangements are made for it… but these other children, who need it quite as much, have it by fits and starts as they can get it, often not in a good or healthy form…’.^96^ The consequence, the author suggested, was that ‘when they are turned loose in a playground, they do not know what to make of it’ and the ‘rougher ones, who have not been kept out of the street, begin to enjoy themselves in their fashion, cuffing each other, shouting and frightening the timid.’ For the author, the answer lay in employing young women who could ‘teach those children… how to play.’ The remainder of the essay, served as a call to arms to the city's young middle-class women: ‘There are many playgrounds in want of workers just now, and more would be opened if persons were willing to take children into them and superintend them.’^97^ While it is almost impossible to know, it would be surprising given the location of the playgrounds and the involvement of so many young women in such ameliorating work if this call went unheeded.

## Conclusion

The MPGA and other such organisations were not always successful in their ambitions. Some of the opposition that they encountered was economic; with owners and/or trustees of sought-after sites seeking levels of financial reward that these philanthropically funded bodies could not afford. Additionally, the issues discussed above relating to the nature of the city's inhabitants and especially the character of its young ‘roughs’ presented a significant barrier to the conversion of some locations. Yet, the discourse of health and moral improvement articulated by the open spaces movement did have a significant impact on the remaking of urban space. Reflecting back on the Association's achievement's at the turn of the century, Lord Brabazon announced that the original objectives of the MPGA had always been kept in view: ‘every suitable opportunity for adding to the open spaces of London, for preserving and improving existing recreation grounds, for planting trees and placing seats in public sites, for assisting the teaching of gymnastics in playgrounds and halls, and for amending the laws relating to open space questions, has been seized.’^98^ Beyond its immediate impact, Brabazon was also keen to highlight what he regarded as the ‘indirect influence’ of the Association. Pointing to the ‘systematic effort being made to provide breathing-spaces for the people,’ which he noted began with Sir Edwin Chadwick and was continued by the CPS and the Kyrle Society, Brabazon stated that the MPGA had been ‘singularly successful in gaining approval and support’ and, by its ‘quiet, constant work,’ had ‘educated the opinion of individuals and the views of the public authorities.’^99^

Based on the evidence presented in this paper, it would be hard to argue against Brabazon's conviction that the MPGA, as well as the Kyrle Society and others, were extremely successful in putting these particular sanitary ideas into practice. This is an important point to acknowledge because we are not dealing here with the grand visions of key figures within the sanitary science movement but rather with the kinds of everyday interventions that were put into effect by concerned citizens who, for a variety of reasons, assembled around this problem. Moreover, focussing on London's disused burial grounds and cemeteries, especially after at least a part of their commercial value was removed with the passing of the Act in 1884, was an inspired tactic. These were sites that would have held an ambiguous place within the minds of the city's inhabitants; as the paper has demonstrated, Brabazon was not alone in describing them as ‘howling wildernesses, filled with rank vegetation, dead men's bodies, yawning graves, and the refuse and garbage of a large city. They are sources of danger to public health and public morals, for here in the darkness, free from police supervision, collects the filthiest human scum which ever shunned the light.’^100^

Most importantly, however, the disused burial grounds and cemeteries were proximate to those who were argued to be most in need and they could be put to the ameliorating work desired of them at only a relatively small cost and with the minimum amount of labour. As discussed above, an important element here was the idea that ‘healthy bodies and compliant minds would be produced, fostered and sustained, if only their surrounding could be made well-ordered, sanitary, pleasant and beautiful.’^101^ Yet, what we witness in this discourse is more than this suggestion that the ‘virtuous social forces’ of certain spaces were amplified so as to ‘reduce those which were damaging or destructive to human health and morality.’^102^ As this paper suggests, while it was recognised that the immanent properties of open, green space would help improve the physical and moral health of those who utilised them through the promotion of good habits it was also recognised that these ameliorating qualities would only be forthcoming if the spaces were kept in order by figures of authority such as the park keeper and the habits of those within them cultivated. There was, then, a disciplinary logic at play in these spaces as well as a vitalist one.

## Footnotes

1 J. London, *The People of the Abyss*, Norwood, 1903, 61.

2 London, *The People of the Abyss* (note 1), 61.

3 London, *The People of the Abyss* (note 1), 62.

4 G. Stedman Jones, *Outcast London*, London, 1971; A.S. Wohl, *The Eternal Slum: Housing and Social Policy in Victorian London*, London, 1977.

5 D. Reeder, Representations of metropolis: descriptions of the social environment in Life and Labour, in: D. Englander, R. O'Day (Eds), *Retrieved Riches: Social Investigation in Britain 1840–1914*, Aldershot, 1995, 323.

6 B. Holmes, Open spaces, gardens, and recreation grounds, in: The Royal Institute of British Architects, *Transactions: Town Planning Conference, London 10–15 October 1910*, London, 1911, 478–498.

7 T. Osborne and N. Rose, Governing cities: notes on the spatialisation of virtue, *Environment and Planning D: Society and Space* 17 (1999) 741; see also F. Driver, *Geography Militant: Cultures of Exploration and Empire*, Oxford, 2001, 170*ff*.

8 This was drawn to my attention by an anonymous referee and is also noted in A. Adams, *Architecture in the Family Way: Doctors, Houses, and Women, 1870–1900*, Montreal, 1996, 11.

9 R. Brabazon, A plea for public playgrounds, *The Quiver* 20 (1885) 338.

10 Brabazon, A plea for public playgrounds (note 9), 336.

11 On the work of these organizations: S.M. Gaskell, Gardens for the working class: victorian practical pleasure, *Victorian Studies* Summer (1980) 480; H.L. Malchow, Public gardens and social action in late-Victorian London, *Victorian Studies* Autumn (1985) 97–124; D. Reeder, The social construction of green space in London prior to the Second World War, in: P. Clark (Ed), *The European City and Green Space: London, Stockholm, Helsinki and St. Petersburg, 1850–2000*, Aldershot, 2006, 31–67; P. Thorsheim, The corpse in the garden: burial, health, and the environment in Nineteenth-Century London, *Environmental History* 16 (2011) 38–68; C. Hickman, ‘To brighten the aspect of our streets and increase the health and enjoyment of our city’: The National Health Society and urban green space in late-nineteenth century London, *Landscape and Urban Planning* (forthcoming). On the influence of the Romantic Movement: P. Macnaghten and J. Urry, *Contested Natures*, London, 1988.

12 H. Meller, *Leisure and the Changing City, 1870–1914*, London, 1976; H. Conway, *People's Parks: The Design and Development of Victorian Parks in Britain*, Cambridge, 1991.

13 F.H.A. Aalen, Lord Meath, city improvement and social imperialism, *Planning Perspectives* 4 (1989) 127–152; Driver, *Geography Militant* (note 7); D. Gilbert, London of the future: the metropolis reimagined after the Great War, *Journal of British Studies* 43 (2004) 91–119.

14 Driver's engagement with Brabazon relates mainly to a discussion of a collection of essays brought together in R. Brabazon, *Social Arrows*, London, 1886 and Gilbert's to a discussion of his contribution to A. Webb (Ed) *London of the Future*, London, 1921; the latter of which makes no reference to his work prior to 1900.

15 These spaces are referred to as disused burial grounds and cemeteries based on the interpretation offered in the Disused Burial Grounds Act, 1884 which states ‘“disused burial ground” means any burial ground which is no longer used for interments, whether or not the ground has been partially or wholly closed for burials under the provisions of a statute or Order in Council.’ 1884 c. 72 (Regnal. 47 and 48 Vict).

16 M. Foucault, The politics of health in the eighteenth century, in: P. Rabinow (Ed), *The Foucault Reader: An Introduction to Foucault's Thoughts*, London, 1984, 273–289.

17 M. Foucault, Space, knowledge, and power, in: P. Rabinow (Ed), *The Foucault Reader: An Introduction to Foucault's Thoughts*, London, 1984, 239–256. See also: J.W. Crampton and S. Elden (Eds), *Space, Knowledge and Power: Foucault and Geography*, Aldershot, 2007.

18 M. Foucault, *Security, Territory, Population: Lectures at the Collège de France, 1977–1978*, Basingstoke, 2007. See also: G. Burchell, C. Gordon and P. Miller (Eds), *The Foucault Effect: Studies in Governmentality*, Chicago, 1991; M. Dean, *Governmentality: Power and Rule in Modern Society*, London, 1999.

19 P. Joyce, *The Rule of Freedom: Liberalism and the Modern City*, London, 2003, 65.

20 On public health and moral topography, see: F. Driver, Moral geographies: social science and the urban environment in mid-century England. *Transactions of the Institute of British Geographers*, N.S. 13 (1988) 275–287; G. Kearns, Biology, class and the urban penalty, in: G. Kearns, C.W.J. Withers (Eds), *Urbanising Britain: Essays on Class and Community in the Nineteenth Century*, Cambridge, 1991, 12–30.

21 Osborne and Rose, Governing cities (note 7), 742.

22 B.W. Richardson, *Hygiea: A City of Health*, London, 1875; see M. Huxley, Spatial rationalities: order, environment, evolution and government, *Social & Cultural Geography* 7 (2006) 771–787; D. Pinder, *Visions of the City*, Edinburgh, 2005.

23 M. Ogborn and C. Philo, Soldiers, sailors and moral locations in nineteenth-century Portsmouth, *Area* 26 (1994) 221–331; Driver, Moral geographies (note 20); see also P. Clark and J.S. Jauhiainen, Introduction, in: P. Clark (Ed), *The European City and Green Space: London, Stockholm, Helsinki and St. Petersburg, 1850–2000*, Aldershot, 2006, 1–29.

24 D.A. Reeder, London and green space, 1850–2000: an introduction, in: P. Clark (Ed), *The European City and Green Space: London, Stockholm, Helsinki and St. Petersburg, 1850–2000*, Aldershot, 2006, 30–40; Osborne and Rose, Governing cities (note 7), 741.

25 Gilbert, London of the future (note 13), 110.

26 Cited in S. Elden, Strategy, medicine and habitat: Foucault in 1976, in: J.W. Crampton, S. Elden (Eds), *Space, Knowledge and Power: Foucault and Geography*, Aldershot, 2007, 73.

27 Osborne and Rose, Governing cities (note 7), 743.

28 The archival work was funded by a travel grant received by the author from the Wellcome Trust. The archives of the MPGA are held at the London Metropolitan Archives. Additional archival material was traced to the Bodleian Library, Oxford University.

29 E. Yeo, *The Contest for Social Science: Relations and Representations of Gender and Class*, London, 1996, 4.

30 Yeo, *Contest* (note 29), 95*ff*.

31 C. Hamlin, *Public Health and Social Justice in the Age of Chadwick*, Cambridge, 1998, 164.

32 Driver, Moral geographies (note 20), 277.

33 Osborne and Rose, Governing cities (note 7), 737.

34 G. Rosen, *A History of Public Health*, Baltimore, 1998 [1958].

35 G. Kearns, Zivilis or Hygaeia: urban public health and the epidemiologic transition, in: R. Lawton (Ed) *The Rise and Fall of Great Cities*, London, 96–124.

36 Hamlin, *Public Health* (note 31), 220.

37 C. Hamlin, Muddling in Bumbledon: on the enormity of large sanitary improvements in four British towns, 1855–1885, *Victorian Studies* 32 (1988) 55–83; S. Szreter, The population health approach in historical perspective, *American Journal of Public Health* March (2003) 421–431.

38 Hamlin, *Public Health* (note 31), 220.

39 Driver, Moral geographies (note 20), 276; Hamlin, *Public Health* (note 31), 249*ff*; R. Paterson, The Health of Towns Association, 1844–1849, *Bulletin of the History of Medicine* 22 (1948) 373–402.

40 Hamlin, *Public Health* (note 31), 252.

41 H.W. Fraser, From civic gospel to municipal socialism, in: D. Fraser (Ed), *Cities, Class and Communication: Essays in Honour of Asa Briggs*, New York, 1990, 58–86.

42 Reeder, London (note 24), 31*ff*.

43 Gaskell, Gardens (note 11), 480; M. Billinge, A time and place for everything: an essay on recreation, re-Creation and the Victorians, *Journal of Historical Geography* 22 (1996) 443–459.

44 M.J. Vernon, On public parks and gardens, *The Sanitary Record* November (1878) 321–322.

45 Driver, Moral geographies (note 20), 278*ff*.

46 Gaskell, Gardens (note 11), 481.

47 O. Hill, Space for the people, *Macmillan's Magazine* 32 (1875) 328.

48 Hill, Space for the people (note 47), 329.

49 A.S. Wohl, *Endangered Lives: Public Health in Victorian Britain*, London, 1983, 178.

50 *The Graphic*, September 9 (1876) 242.

51 H. Frumkin, Healthy places: exploring the evidence, *American Journal of Public Health* 93 (2003) 1451–1456; J. Maas, R. Verheij, P. Groenewegen, et al., Green space, urbanity, and health: how strong is the relation? *Journal of Epidemiology and Community Health* 60 (2006) 587–592.

52 *The Graphic* (note 50).

53 Osborne and Rose, Governing cities (note 7), 744.

54 Along with members of the CPS and the London solicitor Robert Hunter, Hill played a central role in lobbying successfully for legislation to enable churchyards (under the Metropolitan Open Spaces Act, 1877) and later burial grounds (under the Open Spaces Act, 1881) to be maintained as public recreation spaces by local authorities.

55 Malchow, Public gardens (note 11), 109.

56 The name was changed to the Metropolitan Public Garden Association in April, 1885. It is possible the change in name reflected some refocusing of the Association's work following discussions with the Open Spaces Committee of the Kyrle Society.

57 R. Brabazon, *Memories of the Nineteenth Century*, London, 1923, 224.

58 Yeo, *Contest* (note 29), 129.

59 MPGA, *Minutes*, November 1882.

60 Aalen, Lord Meath (note 13), 127. On physicality see: I. Zweiniger-Bargielowska, The culture of the abdomen: obesity and reducing in Britain, circa 1900–1939, *Journal of British Studies* 44 (2005) 239–273.

61 R. Brabazon, Health and physique of our populations, *The Nineteenth Century* 10 (1881) 80–89.

62 Brabazon, Decay of bodily strength in towns, *The Nineteenth Century* 123 (1887) 673–676.

63 Brabazon, Health and physique (note 61), 82.

64 MPGA, *3rd Annual Report* (1885) 32.

65 MPGA, *11th Annual Report* (1893) 8.

66 These figures are taken from the notes that accompanied the map presented in Fig. 3.

67 MPGA, *Minutes* (note 59).

68 Malchow, Public gardens (note 11), 107. Interestingly, Brabazon notes in his memoirs on the nineteenth century that his wife Jeannie, who was at least for a while a member of the Kyrle Society, funded the laying out of a churchyard in Hoxton as a public garden in 1881. See Brabazon, *Memories* (note 57), 222.

69 MPGA, *Minutes* (note 59).

70 Malchow suggests that the MPGA operated as a ‘watch-dog lobby’ at this time. Malchow, Public gardens (note 11), 114.

71 Malchow, Public gardens (note 11), 114.

72 MPGA, *Minutes*, June 1885.

73 MPGA, *Minutes*, December 1890.

74 H.A. Temple, Interview. Miss Wilkinson, *The Women's Penny Paper* 107 (1890) 33–34.

75 J.J. Sexby, *The Municipal Parks, Gardens, and Open Spaces of London. Their History and Associations*, London, 1905.

76 Sexby, *Municipal Parks* (note 75), 574.

77 All of the gardens laid out by the MPGA had official opening ceremonies such as the one at Meath Gardens and most were opened by either aristocrats or other notable figures of the day.

78 *Penny Illustrated* 1894.

79 Meath, The London County Council and open spaces, *The New Review* 7 (1892) 707.

80 W. Besant, The social wants of London, *Pall Mall Gazette* March 18 (1890) 1–2.

81 Besant, Social wants (note 80), 1.

82 Meath, Public playgrounds for children, *The Nineteenth Century* 34 (1893) 269. See also O. Hill, The open spaces of the future, *The Nineteenth Century* 46 (1899) 26–35.

83 Osborne and Rose, Governing cities (note 7), 743.

84 MPGA, *3rd Annual Report*, 12–13.

85 Besant, Social wants (note 80), 1.

86 Joyce, *Rule* (note 19), 10–11.

87 MPGA, *Minutes*, July 1887.

88 MPGA, *Minutes*, July 1884.

89 Meath, Public (note 82), 270–271.

90 E. Gagen, Measuring the soul: psychological technologies and the production of physical health in Progressive Era America, *Environment and Planning D: Society and Space* 24 (2006) 827–849.

91 See Meath, Physical education, *National Review* 20 (1892) 461–467.

92 MPGA, *Minutes*, March 1886; For a discussion of influence of Phillips and Horsfall see C. O'Reilly, From ‘the people’ to ‘the citizen’: the emergence of the Edwardian municipal park in Manchester, 1902–1912, *Urban History* 40 (2013) 136–155.

93 MPGA, *Minutes*, July 1891.

94 Gagen, Measuring (note 90), 835.

95 E. Gagen, Playing the part: Performing gender in America's playgrounds, in: S. Holloway, G. Valentine (Eds), *Children's Geographies: Playing, Living, Learning*, London, 213–229.

96 B. Holland, London playgrounds, *Macmillan's Magazine* 46 (1882) 321–324.

97 Holland, London playgrounds (note 96), 323.

98 MPGA, *18th Annual Report* (1900) 9–10.

99 MPGA, *18th Annual Report* (note 98), 10–11.

100 Meath, The London County Council (note 79).

101 Huxley, Spatial rationalities (note 22), 774.

102 Osborne and Rose, Governing cities (note 7), 745.

